# The roles of long noncoding RNAs in atrial fibrillation

**DOI:** 10.1016/j.ncrna.2023.08.004

**Published:** 2023-08-07

**Authors:** Ozal Beylerli, Jiaming Ju, Aferin Beilerli, Ilgiz Gareev, Alina Shumadalova, Tatiana Ilyasova, Yunlong Bai, Baofeng Yang

**Affiliations:** a Department of Pharmacology (State-Province Key Laboratories of Biomedicine-Pharmaceutics of China, Key Laboratory of Cardiovascular Research, Ministry of Education), College of Pharmacy, Harbin Medical University, Harbin, 150081, China; b Translational Medicine Research and Cooperation Center of Northern China, Heilongjiang Academy of Medical Sciences, Harbin Medical University, Harbin, 150081, China; c Department of Obstetrics and Gynecology, Tyumen State Medical University, 54 Odesskaya Street, 625023, Tyumen, Russia; d Central Research Laboratory, Bashkir State Medical University, Ufa, Republic of Bashkortostan, 3 Lenin Street, 450008, Russia; e Department of General Chemistry, Bashkir State Medical University, Ufa, Republic of Bashkortostan, 3 Lenin Street, 450008, Russia; f Department of Internal Diseases, Bashkir State Medical University, Ufa, Republic of Bashkortostan, 450008, Russia

**Keywords:** Atrial fibrillation, Long noncoding RNA, Structural remodeling, Electrical remodeling, Renin-angiotensin system, Energy metabolism abnormality, Abnormal calcium regulation, Autonomic nerve remodeling

## Abstract

Atrial fibrillation (AF) is a common cardiac arrhythmia that often occurs in patients with structural heart disease and is a significant cause of morbidity and mortality in clinical settings. AF is typically associated with significant changes of both the structure of the atria and the cardiac conduction system. AF can result in reduced heart function, heart failure, and various other complications. Current drug therapy for AF patients is often ineffective and may have adverse effects. Radiofrequency ablation is more effective than traditional drug therapy, but this invasive procedure carries potential risks and may lead to postoperative recurrence, limiting the clinical benefits to some extent. Therefore, in-depth research into the molecular mechanisms of AF and exploration of new treatment strategies based on research findings are prerequisites for improving the treatment of AF and the associated cardiac conditions. Long noncoding RNAs (lncRNAs) are a new class of noncoding RNA (ncRNAs) with a length exceeding 200 nt, which regulate gene expression at multiple levels. Increasing evidence suggests that lncRNAs participate in many pathological processes of AF initiation, development, and maintenance, such as structural remodeling, electrical remodeling, renin-angiotensin system anomalies, and intracellular calcium deregulation s. LncRNAs that play key roles in structural and electrical remodeling may become molecular markers and targets for AF diagnosis and treatment, respectively, while lncRNAs critical to autonomic nervous system remodeling may bring new insights into the prognosis and recurrence of AF. This review article provides a synopsis on the up-to-date research findings relevant to the roles of lncRNAs in AF.

## 1 Introduction

Atrial fibrillation (AF) is one of the most common clinical arrhythmias, which can cause serious complications such as stroke and heart failure, making it a major threat to human health. Epidemiological surveys show that there are approximately 8 million AF patients in China, with an AF incidence rate of about 0.71% in individuals over the age of 35 [1]. Even worse is that AF incidence rate is on the rise in overall population and more so with increasing age, reaching as high as 2.35% in individuals over 75 years old. This not only increases medical costs for patients and reduces their quality of life, but also places a huge burden on the country's healthcare system.

The treatment goal for AF is to improve symptoms and prevent complications, mainly achieved through three approaches: (1) anticoagulant therapy with thrombolytic agents to minimize the risk of, or prevent, stroke; (2) controlling heart rhythm by restoring or maintaining sinus rhythm; and (3) controlling heart rate to prevent the occurrence of rapid ventricular rate. These treatment procedures, though optimized based on updated knowledge and technologies, often have poor efficacy and potential adverse reactions. Therefore, it is imperative to advance our understanding of the pathogenesis of AF, discover new targets for intervention, and improve the shortcomings of current AF treatment regimens.

Long noncoding RNAs (lncRNAs) belong to a class of noncoding RNA (ncRNAs) with a length exceeding 200 nt. They are transcribed by RNA polymerase II and undergo polyadenylation and splicing. LncRNA genes have their own promoter structures and distribute widely throughout the genome [2]. In recent years, numerous studies have demonstrated that lncRNAs not only participate in the development of cardiovascular diseases such as atherosclerosis, hypertension, coronary heart disease, cardiomyopathy, and heart failure, but also play critical roles in many pathological processes of atrial fibrillation (AF) development, such as structural remodeling, electrical remodeling, renin-angiotensin system (RAS) anomalies, and intracellular calcium handling deregulation, among others [[3], [4], [5], [6]].

## 2 Classification and functions of LncRNAs

**Fig. 1:**
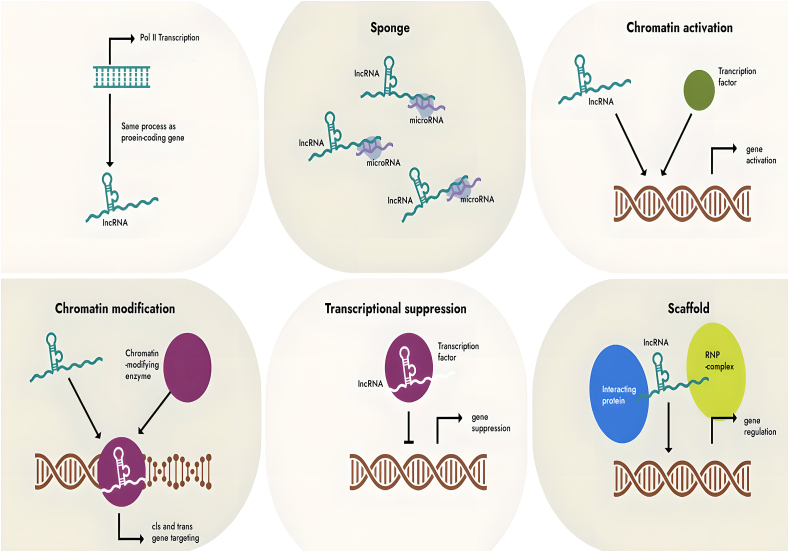
Is a cartoon diagram that visually explains the process of biogenesis and classification of long noncoding RNAs (lncRNAs) in humans and other animals. It outlines how lncRNAs are categorized based on their cellular localization and the specific mechanisms involved in their generation. The diagram provides an illustrative representation of the complex regulatory roles and diverse functions of lncRNAs within cells.

Based on the position relationship between lncRNA and its adjacent protein-coding gene, it can be roughly divided into 5 categories, including sense lncRNA, antisense lncRNA, bidirectional lncRNA, intronic lncRNA, and intergenic lncRNAs [7]. Sense lncRNA is transcribed from the sense strand of a protein-coding gene and can overlap one or more exons of protein-coding genes on the same strand; antisense lncRNA is transcribed from the antisense strand of a protein-coding gene; bidirectional lncRNA is transcribed in the opposite direction of protein-coding genes, and the distance between them is generally less than 1000 base pairs; intronic lncRNA comes from the introns of protein-coding genes and does not overlap with any exons; intergenic lncRNA is located in between two genes, with independent transcription units but without overlap with protein-coding genes [8]. In addition, genome-wide studies have shown that enhancers can also be transcribed, giving rise to a new type of lncRNA, enhancer-derived RNA (eRNA) (Fig. 1) [9].

**Fig. 2:**
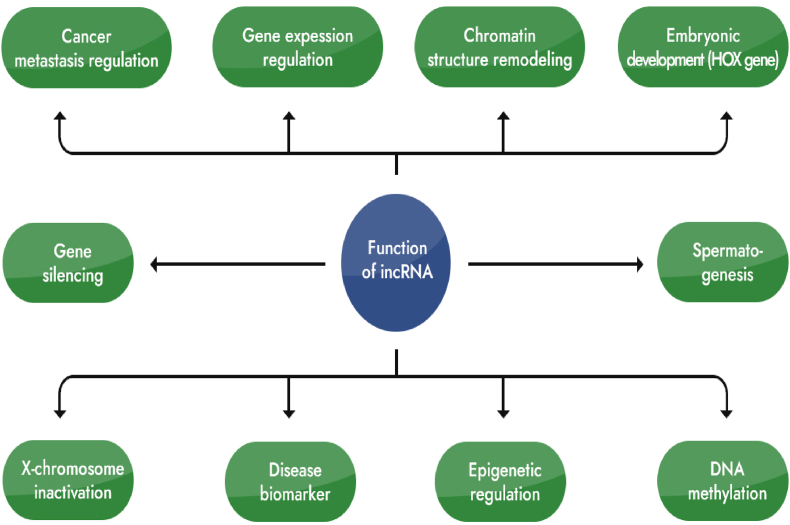
Presents a diagram that visually showcases the various roles played by long noncoding RNAs (lncRNAs) in a wide range of biological processes. The figure illustrates how lncRNAs participate in diverse cellular activities, highlighting their multifunctional nature and significant contributions to various aspects of cellular regulation and function.

The biological functions of lncRNAs mainly include three aspects: 1) lncRNAs can regulate gene transcription in the nucleus. For example, lncRNAs can directly silence the transcription of nearby genes by regulating histone H3 methylation through the *cis*-regulatory pathway; on the other hand, lncRNAs can activate or suppress gene expression of distant gene loci on different chromosomes through the *trans*-regulatory pathway. In addition, eRNA can directly act on enhancers and regulate their activity (Fig. 2) [[10], [11], [12]]. 2) lncRNAs participate in post-transcriptional regulation in the cytoplasm, promoting or inhibiting mRNA translation, changing mRNA and protein stability, and even altering protein localization. They can also act as competitive endogenous RNAs (also known as microRNA sponges) by directly binding to miRNAs to regulate the expression of their downstream target genes [13]. For example, lncRNA MALAT1 can target miR-200 to regulate H_2_O_2_-mediated oxidative damage in cardiomyocytes [[14]a, [14]b]. 3) lncRNAs can also exert biological effects through the exosome pathway. LncRNAs can be packaged in vesicles such as exosomes and secreted into the extracellular space via protein binding or without them [15].

## 3 LncRNAs dysregulation in AF

**Fig. 3:**
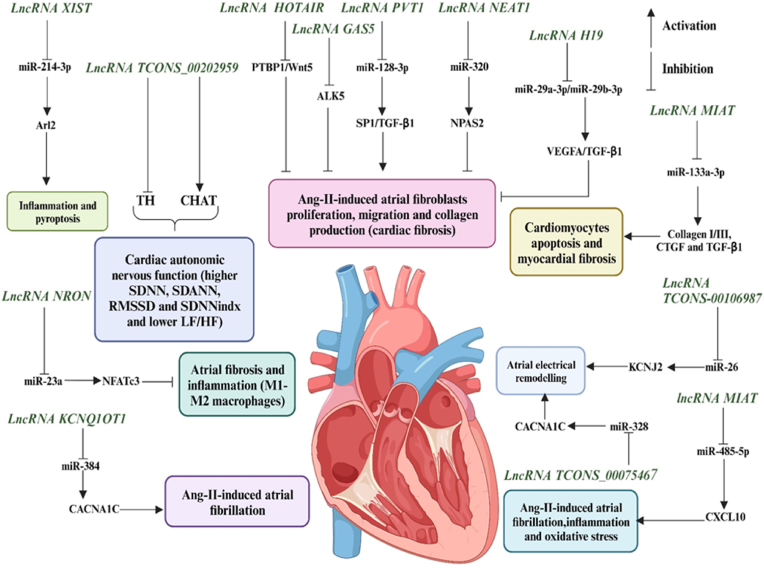
Schematic illustration of some studies in vitro and in vivo on the study of regulatory activity of long non-coding RNAs (lncRNAs) in atrial fibrillation (AF). The activity of lncRNAs in AI is based on a fine line of regulation of complex molecular networks at the level of epigenetic changes, transcription, and post-transcriptional transformations, such as lncRNA/microRNA (miRNA)/mRNA connections, which is necessary for the control of proliferation and migration of cardiomyocytes, collagen synthesis, inflammation, atrial electrical remodeling, cardiac autonomic nervous function, and oxidative stress. Note: Ang-II, Angiotensin II; XIST, X-inactive specific transcript; HOTAIR, HOX antisense intergenic RNA; PVT1, Plasmacytoma variant translocation 1; GAS5, Growth arrest-specific 5; NEAT1, Nuclear paraspeckle assembly transcript 1; MIAT, Myocardial infarction associated transcript; NRON, Non-coding repressor of NFAT; KCNQ1OT1, KCNQ1 opposite strand/antisense transcript one gene; Arl2, ADP ribosylation factor like GTPase 2; TH, Tyrosine hydroxylase; CHAT, Choline acetyltransferase; NFATc3, Nuclear factor of activated T cells 3; CACNA1C, Calcium voltage-gated channel subunit alpha1 C; PTBP1, Polypyrimidine tract-binding protein 1; Wnt5, Wnt oncogene analog 5; ALK5, Activin receptor-like kinase 5; nSP1, Specificity protein 1; nTGF-β1, Transforming growth factor-beta 1; NPAS2, Neuronal PAS domain protein 2; VEGFA, Vascular endothelial growth factor A; CTGF, Connective tissue growth factor; KCNJ2, Potassium inwardly rectifying channel subfamily J member 2; CXCL10, C-X-C motif chemokine ligand 10; SDNN, Standard deviation of NN intervals; SDANN, SDNN of atrial; RMSSD, Root mean square of successive differences; SDNNindx, SDNN intervals in all 5-min segments; LF, Low frequency; HF, High frequency.

LncRNAs are differentially expressed in AF. Wu et al. investigated the expression profile of lncRNA in the atria of patients with rheumatic mitral valve disease and found 16 differentially expressed lncRNAs relative to controls [16]. Among them, lncRNA n336928 may participate in the pathogenesis of AF by regulating fibrosis-related proteins such as Smad2, TGF-β1, matrix metallopeptidase 9 (MMP9), and tissue inhibitor of metalloproteinase 1 (TIMP1), but its specific expression and functional regulation need further study. Ruan et al. used microarray technology to detect the expression profile of lncRNAs in the atrial tissue of AF patients and compared it with non-AF patients and identified 219 differentially expressed lncRNAs [17]. The authors then selected 5 upregulated and 5 downregulated lncRNAs for real-time quantitative PCR validation and confirmed that 4 of them were associated with AF-related genes. Similarly, Xu et al. found t 177 differentially expressed lncRNAs in AF patients relative to controls and predicted, by constructing a co-expression network, that these lncRNAs were regulated by transcriptional regulatory elements, including nucleosome transcription factor 1, TATA-binding protein-associated factor, and early B cell factor [18]. In addition, Ke et al. found differential expression of lncRNAs in the left and right atria of AF patients [19]. Chen et al. compared the lncRNAs in the left atrial appendage to pulmonary vein circumferential and left atrial tissue using a gene chip and identified 94 differentially expressed lncRNAs, with lncRNA AK055347 showing the most significant changes (Fig. 3) [20].

## 4 The role of lncRNAs in structural remodeling in AF

Structural remodeling associated with AF is characterized by abnormal proliferation of fibroblasts and excessive deposition of extracellular matrix leading to atrial fibrosis [21]. The TGF-β1/Smad pathway is the most common mechanism for atrial fibrosis, and upregulation of TGF-β1 can promote atrial fibrosis thereby AF [22]. Zhao et al. analyzed the adipose tissue in the epicardium and found that the epicardial adipose tissue can secrete multiple lncRNAs, which diffuse passively to adjacent myocardial tissue and regulate atrial remodeling [23]. Cao et al. found that lncRNA PVT1 can bind to miR-128–3p, activate TGF-β1, thereby inducing atrial fibrosis [24]. Overexpression of PVT1 in fibroblasts enhances TGF-β1 signal and increases collagen I and II, while knocking down PVT1 expression produces the opposite effects. Another study revealed that lncRNA MIAT is upregulated in peripheral blood leukocytes of AF patients, and the expression of miR-133a-3p is significantly downregulated, which is consistent with the results obtained from atrial tissue of AF rat models [25]. Their results further demonstrated that MIAT regulated the expression of TGF-β1 and the generation of collagen fibers in the atrium through sponging miR-133a-3p [25].

The type I receptor Alk5 is an important molecule that promotes tumor cell proliferation and regulates the expression of TGF-β1 [26]. It has recently been shown to regulate the activities of cardiac fibroblasts. LncRNA GAS5 can inhibit the proliferation of cardiac fibroblasts by downregulating Alk5 expression, ultimately delaying the development of AF [27]. Macrophages also play a role in myocardial fibrosis, and in general they have two phenotypes, M1 and M2. In response to damage, M1 macrophages are the first to arrive at the site of injury and promote tissue debris clearance, while M2 macrophages play a key role in subsequent tissue repair and healing. Studies have shown that inhibiting M1 macrophages and/or promoting M2 macrophages prevent cardiac remodeling [28]. Sun et al. found that lncRNA NRON (repressor of the nuclear factor of activated T cells) reduced IL-12 expression by inhibiting the nuclear transport of activated transcription factor 1, leading to a decrease in M1 macrophages and ultimately delaying the progression of myocardial fibrosis [29]. In addition, NRON also inhibited fibroblast proliferation by increasing the phosphorylation of activated T cell transcription factor c3, thereby reducing atrial fibrosis [30]. Structural remodeling is an extremely important aspect of AF development. By targeting cardiac fibrosis, lncRNAs can improve primary and secondary prevention of AF and even other cardiovascular diseases [31,32]. However, in addition to the TGF-β1/Smad pathway, the JAK/STAT and PI3K/Akt pathways can also induce myocardial fibrosis [31,32].

## 5 LncRNAs and cardiac electrical remodeling

In AF, cardiac electrical remodeling is mainly characterized by a shortened effective refractory period and action potential duration. Li et al. found that lncRNA-TCONS_00075467 was specifically expressed in a rabbit AF model [33]. Silencing TCONS_00075467 with short hairpin RNA (shRNA)-carrying lentivirus effectively shortened the effective refractory period and action potential duration likely by decreasing atrial L-type calcium channel current. Additionally, TCONS_00075467 adsorbed miR-328 to derepress the downstream target gene L-type voltage-dependent calcium channel subunit α1C expression to alleviate AF electrical remodeling. Several cardiac-specific transcription factors, such as paired-like homeodomain transcription factor 2 (PITX2) and T-Box transcription factor 5 (TBX5), have been shown to be involved in the regulation of ion channel genes and play a role in AF, with decreasing PITX2 level shortening atrial effective refractory period [34,35]. Gore-Panter et al. identified a lncRNA gene localized upstream to the PITX2 gene, called PITX2 adjacent non-coding RNA (PANCR) [36]. The expression of PITX2 and PANCR is positively correlated, and knocking down PANCR synchronously reduces the level of PITX2. Therefore, PANCR likely participates in regulating the occurrence and development of AF through regulating PITX2 expression. It is worth noting that there is no complementary binding site between PANCR and PITX2. Considering the functional characteristic of lncRNA as a miRNA sponge, it is possible that PANCR indirectly regulates PITX2 through targeting miRNA. Yet, the relationship between PANCR and PITX2 needs to be clarified and verified using biological information analysis for target prediction, in conjunction with luciferase reporter gene, RNA immunoprecipitation, and RNA pulldown assays. Deficiency of TBX5 can cause irregular depolarization and atrial conduction slowing, leading to rapid onset of AF [37]. Yang et al. identified a TBX5-dependent lncRNA, RACER, but whether RACER plays a role in AF remains to be clarified [38].

## 6 LncRNAs and RAS

Activation of RAS increases the secretion of angiotensin II (Ang II), which not only increases left atrial pressure, leading to left atrial enlargement, but also alters ion channels on the myocardial cell membrane. Prolonged activation of RAS can further cause fibrosis and inflammation of myocardial tissue [39]. Shen et al. found that lncRNA KCNQ1 overlapping transcript 1 (lncRNA KCNQ1OT1) was significantly upregulated in a mouse model of AF induced by Ang II. KCNQ1OT1 can bind miR-384 and upregulate the expression of L-type voltage-dependent calcium channel α1C subunit, thereby promoting AF development [40]. Ubiquitin carboxyl-terminal hydrolase L1 (UCHL1) can promote Ang II-induced AF through multiple signaling pathways such as AKT, ERK1/2, HIF-1α, and TGF-β/Smad2/3 [41]. A study showed that the translation of UCHL1 protein is regulated by lncRNA UCHL1-AS1 [42]. However, further research is needed to verify whether lncRNA UCHL1-AS1 participates in the occurrence and development of AF by intervening in UCHL1 expression.

## 7 LncRNAs and energy metabolism dysfunction

van Bilsen et al. first proposed that changes of energy metabolism, caused by high-energy phosphate metabolism disorders and mitochondrial dysfunction, occur in heart failure and myocardial hypertrophy [43]. Energy metabolism abnormalities associated with AF mainly manifest as changes in adenosine nucleotide protein kinase, mitochondrial dysfunction, and accumulation of reactive oxygen species [44]. Peroxisome proliferator-activated receptor gamma coactivator 1 alpha and peroxisome proliferator-activated receptor gamma (PGC-1α/PPARγ) are important factors in AF energy metabolism abnormalities. They can improve lipid metabolism in AF by regulating adenosine nucleotide protein kinase and mitochondrial function by deacetylation [45,46]. Li et al. identified the energy metabolism-related lncRNA TCONS_00016478 in AF rabbits by high-throughput screening [47]. Downregulation of this lncRNA decreased PGC-1α/PPARγ levels, leading to lipid deposition in atrial myocardium [47]. However, further research is needed on whether TCONS_00016478 can improve mitochondrial function through the PGC-1α/PPARγ pathway. In addition, lncRNA AK055347 was found significantly upregulated in AF patients, which inhibited mitochondrial energy production by regulating the expression of Cyp450, ATP synthase, and MSS51 [48].

In mice with diabetic nephropathy, lncRNA Tug1 functioned as an eRNA to promote the activation of PGC1-α, improving mitochondrial function in foot cells [48]. However, it is not clear whether Tug1 is expressed in AF. Li et al. found that lncRNA HOTAIR bound and inhibited miR-125 to derepress its downstream target gene matrix metallopeptidase 2 (MMP2) and aggravate oxidative stress-induced myocardial injury [49]. MMP2 has been shown to be associated with AF [50]. HOTAIR is likely to promote myocardial oxidative stress and accelerate AF development through the miR-125/MMP2 pathway. During AF, the rapid contraction and relaxation of the atrial muscle consumes a large amount of energy. Therefore, maintaining the balanced energy metabolism in the atria may be a new therapeutic approach for AF, but the studies on energy metabolism disorders in AF have been sparse and future in-depth research on this issue is needed.

## 8 LncRNAs and calcium dysregulation

Calcium dysregulation is related to calcium storage and release in cardiomyocytes, which is dependent on sarcoplasmic reticulum calcium ATPase 2a (SERCA2a) and ryanodine receptor 2 (RyR2). SERCA2a has been shown to inhibit the occurrence of AF [51]. In a mouse model of myocardial infarction, lncRNA ZFAS1 can induce cytoplasmic Ca^2+^ overload and trigger mitochondria-mediated apoptosis by inhibiting SERCA2a [52]. In addition, lncRNA DACH1 was found to promote the degradation of SERCA2a through ubiquitination, exacerbating heart dysfunction [53]. Although there is currently no evidence that these lncRNAs are directly associated with AF, given their modes of actions, these lncRNAs are likely important in AF.

RyR2 is regulated by Junctophillin-2 (JP2), a signaling protein involved in sarcoplasmic reticulum coupling. A clinical study showed that JP2꞉RyR2 values were decreased in AF patients, accompanied by downregulation of lncRNA-LINC00472 and upregulation of miR-24 [54]. MiR-24 can reduce JP2 expression, while lncRNA-LINC00472 can inhibit miR-24 and increase JP2꞉RyR2 values, ultimately improving AF.

## 9 LncRNAs and autonomic nerve remodeling

Autonomic nerve remodeling leading to neural sprouting and imbalance between sympathetic and parasympathetic nervous systems is an important mechanism for AF [55]. Research using sequencing and bioinformatics analysis has found abnormal expression of lncRNAs in the cardiac fat pad of dogs with AF, and these lncRNAs (such as lncRNA TCONS_00032546 and TCONS_0002610) are related to autonomic nerve remodeling [56]. Growth associated protein-43 (GAP-43) is an important marker for neural growth or regeneration, while tyrosine hydroxylase (TH) is the limiting enzyme in the synthesis of sympathetic neurotransmitters, and its expression indicates the distribution of sympathetic nerves in the heart [57]. Overexpression of lncRNA056298 after radiofrequency ablation in dogs increased GAP-43 and TH levels, shortened atrial effective refractory period, and increased AF inducibility, indicating that lncRNA056298 may promote AF by mediating neural remodeling through GAP-43 and TH [58]. In addition, Zhao et al. found that lncRNA TCONS_00202959 was downregulated in AF rats, which was accompanied by shortened atrial effective refractory period and increased AF inducibility, and subsequent analysis of heart rate variability pointed to autonomic dysfunction [59]. After transfection with lncRNA TCONS_00202959 overexpressing lentivirus, atrial effective refractory period in AF rats was prolonged, AF inducibility was decreased, autonomic nerve function was significantly improved, and TH levels were decreased, indicating that lncRNA TCONS_00202959 may inhibit the occurrence and development of AF by improving cardiac autonomic nerve function. The period of autonomic nerve remodeling is relatively long, and we believe that it could be a marker for predicting AF recurrence.

## 10 Conclusions

**Fig. 4:**
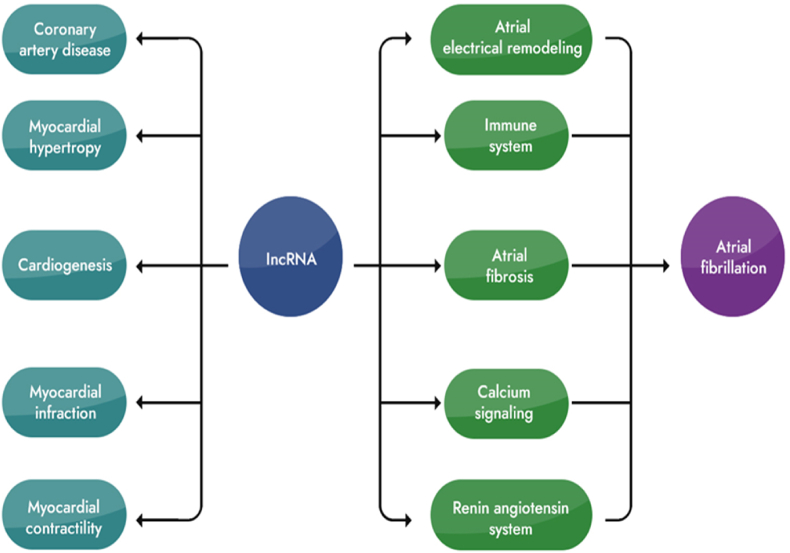
The diagram illustrates the involvement of long noncoding RNAs (lncRNAs) in cardiovascular disease and atrial fibrillation (AF). These lncRNAs play crucial roles in different aspects of cardiovascular pathophysiology, including coronary artery disease, myocardial hypertrophy, myocardial contraction, myocardial infarction, and heart organogenesis. Moreover, they contribute significantly to the development of AF by influencing atrial fibrosis, calcium signaling, electrical remodeling, as well as the renin-aldosterone system and the immune system. The diagram visually represents how lncRNAs impact these critical processes, highlighting their importance as potential targets for therapeutic interventions in cardiovascular diseases and AF.

Long noncoding RNAs (lncRNAs) exert their regulatory influence on gene expression at multiple levels, encompassing epigenetic, transcriptional, translational, and post-translational modifications [[60], [61], [62], [63]]. In the context of atrial fibrillation (AF) development (as depicted in Fig. 4), lncRNAs play critical roles in structural, electrical, neural, and energy metabolic remodeling, among other key processes.

Despite these promising observations, our current understanding of the relationship between lncRNAs and AF remains at an early stage, limiting their full application in clinical diagnosis, prognosis, and treatment (summarized in Table 1).

**Table 1:** The role of lncRNAs in the development and pathophysiological remodeling of AF.

Development of AF				
LncRNA	**Expression in AF**	**Mechanism of Action**	**Study Model**	**Reference**
**AK055347**	Up	dysregulation of mitochondrial energy production can occur through the regulation of mitochondrial Cyp450, ATP synthase, and MSS51, leading to alterations in their functioning	Patient (AF)	[20]
**RP3-523K23.2**	ND	It is possible that their involvement in atrial fibrillation (AF) occurs through the modulation of HSF2 transcription	Patient (AF)	[64]
**RP11**–**99E15.2**	ND	Their potential role in atrial fibrillation (AF) could be attributed to their ability to regulate the binding of extracellular matrix through interactions with ITGB3	Patient (AF)	[64]
Structural Remodeling				
**PVT1**	Up	By acting as a miR-128–3p sponge, they potentially regulate the miR-128–3p/Sp1/TGF-β1/Smad axis in atrial fibrillation (AF)	Patient (AF) or Atrial fibroblast; Mouse heart (Ang-II)	[24]
**MIAT**	Up	They potentially mitigate atrial fibrillation (AF) and decrease atrial fibrosis by inhibiting the expression of miR-133–3p	Patient (AF); Rat (electrical stimulation)	[25]
**GAS5**	Down	By inhibiting ALK5, they potentially suppress cell proliferation	Patient (AF) or AC16	[27]
**NRON**	Up	By preventing the localization of NFAT to the nucleus, they potentially hinder the activation of IL-12 and inhibit the macrophage transition from an M2 to an M1 phenotype	Mouse atrial CM (AngII)	[29,30]
**TCONS_00032546**	Down	They are associated with the remodeling of neurons in cardiac fat pads mediated by the RAS pathway	Canine heart (atrial tachypacing)	[56]
**PCAT1**	Up	By targeting TGF-β1, they potentially enhance fibroblast proliferation	Patient (AF) or AC16	[65]
**TCONS_00026102**	Down	They are potentially involved in the remodeling of neurons in cardiac fat pads through the activation of the RAS pathway	Canine heart (atrial tachypacing)	[66]
Electrical Remodeling				
**TCONS_00075467**	Down	When upregulated, they lead to an increased ability to sponge miR328, resulting in elevated levels of CACNA1C	Rabbit right atria (AF)	[33]
**KCNQ1OT1**	Up	When downregulated, they lead to a decreased capacity to sponge miR384, resulting in reduced levels of CACNA1C	Mouse heart (AngII) or CM	[40]
**NPPA-AS1**	Up	They potentially regulate genes involved in cardiac contraction, such as NPPA, PLCE1, TNNC1, and TNN1	Patient (AF)	[64]
**lncRNA-HBL1**	Up	By reducing the expression of miR-1, a gene associated with atrial fibrillation (AF) but not extensively investigated in AF specifically	Human iPSC-CM	[67]

One potential avenue for clinical application lies in the detection of lncRNAs in blood, which could serve as valuable clinical biomarkers. However, their precise sources, specific functions, and exact relationship with AF remain unknown. Furthermore, their relatively low abundance in blood necessitates time-consuming and costly detection using traditional methods like PCR. To address this, researchers have proposed the use of rolling circle amplification fluorescence detection of lncRNAs, which can amplify lncRNA levels in samples by tens of times. However, this approach has not yet been implemented in clinical settings. Another promising aspect is the high tissue specificity of lncRNAs, which allows them to regulate the development of AF. As a potential strategy for AF therapy, replacement therapy involves restoring or increasing the expression of downregulated or low-abundance anti-AF lncRNAs in tissues to achieve therapeutic effects. Currently, common methods include the delivery of lncRNA-overexpressing plasmids, though their efficiency remains a challenge. Adenovirus infection can be an alternative solution, but it is limited in carrying longer lncRNA fragments. On the other hand, CRISPR gene editing tools have shown potential in transcribing longer lncRNAs into the genome. For loss-of-function therapy, repressing the overexpressed pro-AF lncRNAs critical to the pathogenesis of certain diseases, like AF, is pursued. GapmeR, a cell-permeating antisense single-stranded DNA molecule supporting RNase H cleavage, has shown promise in silencing lncRNAs in both the cytoplasm and the nucleus. While these methods have shown encouraging results in animal models, their translation to humans necessitates rigorous validation due to the poor conservation of lncRNAs across species. Additionally, it remains uncertain whether the same methods will work effectively in the human body. The challenge of organ-, tissue-, and cell-specific delivery of lncRNA constructs to produce atrium-specific effects is another hurdle in their clinical application. Furthermore, the safety profile of lncRNA therapy remains unknown, requiring thorough evaluation of potential adverse effects. Despite the progress in exploring the role of lncRNAs in AF, most studies published to date focus on their associations without fully elucidating the specific molecular mechanisms involved. Consequently, the exact mechanistic implications of lncRNAs in AF development remain elusive, warranting further in-depth research. As our understanding of lncRNA functions and interactions advances, addressing these challenges and knowledge gaps will pave the way for more effective clinical applications of lncRNA-based therapies in the management of AF and cardiovascular diseases.

## Funding

National Natural Science Foundation of China (NSFC; Grant No. 82170240). This work was supported by the Bashkir State Medical University Strategic Academic Leadership Program (PRIORITY-2030).

## CRediT authorship contribution statement

**Ozal Beylerli:** conceptualized and designed the study. All authors have participated in the acquisition, analysis, and interpretation of the data. **Jiaming Ju:** has drafted the manuscript. **Aferin Beilerli:** has drafted the manuscript. **Ilgiz Gareev:** has drafted the manuscript. **Alina Shumadalova:** contributed to the critical revisions of the manuscript. **Tatiana Ilyasova:** contributed to the critical revisions of the manuscript. **Yunlong Bai:** supervised the research. All authors agreed on the journal to which the article would be submitted, gave the final approval for the version to be published, and agreed to be accountable for all aspects of the work. **Baofeng Yang:** supervised the research.

## Declaration of competing interest

Ozal Beylerli is an editorial board member for Non-coding RNA Research and was not involved in the editorial review or the decision to publish this article. All authors declare that there are no competing interests.
